# Diseases of the Respiratory Organs

**Published:** 1905-06-17

**Authors:** 


### DISEASES OF THE RESPIRATORY ORGANS.

**Consumptive Sanatoria.** — C. G. Higginson[1] analyses the writings of Bodington and MacCormac on the cure of phthisis, mentioning by the way Sydenham's advocacy of open air. It is a curious fact that both Bodington and MacCormac, though they never met, published in 1840 treatises in which they laid down almost the exact combination of rules which we now know as the open-air sanatorium method. Both were uncompromising on the need of open air by night and by day and of a good nourishing diet. MacCormac appreciated better the need of rest in febrile conditions and of bodily warmth, and showed how easily consumption may be prevented, while Bodington was more skilful in his treatment of cough and sleeplessness. The teaching that "re-breathed" air is an active cause of consumption was unheeded until the work of Brehmer and Walther and the pathological discoveries of Koch broke down prejudice. In 1854 Brehmer[2] laid down once more that "abundance of the purest air, good food, and a careful regulation of rest and exercise are the three essentials of treatment. Subsequent results have shown that the combination of these three elements does wonderfully increase the resisting power of the organism. W. B. Ransom discusses what answer we should give to anyone who asks the question Are the sanatoria, especially those for the poor, worth their cost? Even from the experience of the last ten years in England there is no doubt of the results attained, and Ransom has no difficulty in proving the value of the method from numerous statistics and the facts of personal observation. It has been pointed out, however, that from a want of uniformity in the reports of different institutions the full value of the statistics is not gained, and that we ought to collect an immense number of records from numerous places all drawn up on exactly the same lines, and that the stages of the disease and the condition of patients before and after treatment should be everywhere stated in the same terms as exactly as possible. It was by such an overwhelming collection of facts that the cold bath treatment was shown beyond doubt to save so many lives in typhoid, but the statistics in phthisis are far more difficult to bring into line. Ransom quotes the records of the German sanatoria for working people as turning out from 68 to 77 per cent. capable of work, but it appears that only 30 per cent. are still capable four years afterwards, though at Basel-Davos that number rises to 58 per cent. In English sanatoria the success seems to be about the same, and he finds that the general result of the method is that 25 to 33 per cent. are fit for work for at least four years. These figures are based on the supposition that there is little selection of cases. When patients are sent at an earlier stage, it is certain that recoveries are far greater. Weicker's record on this point is most striking, though perhaps exceptional. Out of 1,367 patients three years after treatment, 95 per cent. of those in the first stage were at work, and 95 per cent. of those in the third stage were dead. Ransom mentions, indeed, that some of his Nottingham patients who were only "improved," have done remarkably well and were among the most satisfactory cases, but the fact remains that, if a high average of permanent success is to be reached, patients must be sent in at a much earlier stage than at present. This is especially true of working-class patients, where the time spent in sanatoria must be limited, and where the conditions of life afterwards are defective. The direct benefit of the sanatorium is, of course, only one part of the patient's gain; for he is, while there, taught and disciplined in the proper care and management of his own health. The permanency of the cure depends largely upon his own perseverance in carrying on the method afterwards and also upon the more or less favourable hygienic conditions under which he has to live for the next few years. Hence the importance of some arrangement for the after-care of consumptive convalescents to enable them to obtain good food, healthy rooms, and suitable work afterwards. Burton Fanning finds that of early cases 75 per cent. can be restored to lasting fitness for work and 90 per cent. will survive indefinitely if they adhere to their method. Of mixed cases 32 per cent. can be cured and restored to permanent fitness, and in another 25 per cent. the disease will be arrested, but the patients will be unfit for hard work. Trudeau[3] records a permanent cure in 66 per cent. of the incipient cases and 31 per cent. of mixed ones. Thurnam[4] and Wheeler, at Nordrach on Mendip, found that out of 289 patients heard from at least a year after leaving 35·9 per cent. were cured and another 17·6 of arrested cases were in fair health. Of early cases 90·9 per cent. were cured. Large numbers of similar statistics could be added, but enough has been said to show the good results in what was not long ago an almost hopeless disease. Still, there are very numerous cases for whom sanatoria are unavailable. Thus special dispensaries have been started for patients living at home, who are aided in obtaining good food, provided with medicine, and taught how to regulate their lives. W. Ewart[5] thinks that besides these we need (1) simple, inexpensive open-air stations for the earliest and mildest cases; (2) asylums for the hopelessly advanced, and (3) elaborate sanatoria for all other degrees. In the last group he would have arrangements for treating severe cases both by prolonged care and other additional measures, such as formaldehyde and some form of serum. In Germany there were recently 57 sanatoria in operation and 27 building. in France only 30, but in the United States the sanatoria amount to about 200, and there are no less than 53 societies for the suppression of tuberculosis. Hector Mackenzie[6] remarks that under open-air treatment night sweats usually cease, secondary infection from re-inhaled bacilli is less frequent, and the assimilation of food is greatly increased. The capacity of tuberculous patients for putting on flesh is remarkable, but it is desirable to encourage the effort to eat rather in those patients who are actually under-weight than in the well nourished. In the matter of rest, continuous evening temperatures of 101°, or a morning temperature above normal, indicate that the patient should not get up, and a pulse-rate of over 100° should forbid exercise. Rest for half an hour at least before meals, and the careful graduation of exercise are important. If the temperature is raised by more than two degrees after exertion, that exercise clearly is excessive. Treatment at a sanatorium should be kept up for six months or more, and Mackenzie, while he thinks it the best treatment in existence for most patients, strongly protests against sending there advanced or very rapidly-progressing cases. Real dyspepsia in phthisis is rare. Coughing brought on by taking food can often be prevented by 5 grains each of alum and liq. potassæ taken a few minutes before a meal. In apyretic cases which do not improve beyond a certain point under the best treatment, tuberculin may be employed. Pasquier[7] has made certain investigations on digestion in phthisis and finds that in early stages there is hyperchlorhydria, but that later on there is a deficiency of acid. Hence in the early stages cod-liver oil is useful, but such drugs as arsenic, kola, and creosote should be avoided, whereas in later stages stimulants to the glandular activity are needed. R. C. Macfie,[8] after an experience of 5,000 cases under open-air treatment, claims that sanatorium treatment is better than treatment at high altitudes, or in any other way. If sanatoria for the poor are to be of real value two things are necessary—(1) they must receive only early cases; (2) they must provide some home treatment after the patients leave and until they are past all danger of retrogression. As a rule not more than 10 per cent. in sanatoria now are early cases, and the duration of treatment is insufficient if they have to return to work and crowded homes. Thus at the Durham Sanatorium 122 out of 168 were advanced cases, and at Sheffield 39 per cent. were hopeless on admission. At present there are about 1,000 beds for poor patients in this country; 80 per cent. are filled with the wrong patients, the other 20 per cent. are not kept long enough. In his own sanatorium at Sidlaw at first 73 per cent. of the incipient cases showed arrest, and 31 per cent. of the advanced, but when more well-to-do patients were taken the results improved.

W. Gordon[9] gives statistics from Exeter showing that in streets fully exposed to rainy wind the death-rate from phthisis is much higher than in sheltered ones, and that a higher rate is found on clay soils than on gravel. In the most exposed rural districts of Devon the rate is twice as high as in the least exposed. Indeed, from the statistics of other countries as well, he concludes[10] that exposure to rainy wind has more effect than anything else except race and occupation, while rainfall and dry wind by themselves have little or none. Friedmann[11] finds that tubercle bacilli of the turtle are harmless to warm-blooded animals, and that when injected into oxen a single dose renders them immune to fatal doses of bovine bacilli. He thinks that cures can be effected by this method, and points out that the serum of animals treated in this way creates immunity in other animals. T. Dewar[12] reports nine cases treated by his intravenous method of iodoform injections, and considers that in five the disease was arrested. He asserts that the continued injection of an ethereal solution of iodoform in half-grain doses is harmless, and also powerful to arrest tuberculosis. He now adds 40 per cent. pure liquid paraffin to the solution, which is much less irritating than one containing ether alone. Among various authors who report favourably or otherwise of Marmorek's serum we learn that at Brompton in active and progressive cases it was found of little value.[13] Frey,[14] at Davos, found it useful in chronic cases. It is well tolerated, though rashes are frequent. The injections are given under the skin of the upper arm, and are followed generally by a fall of temperature and reduced expectoration—seven or eight doses can be given in 10 days, after which none is given for a similar period. The series is repeated several times in a similar order. Marmorek has treated 2,000 cases with his serum and finds it most useful in those cases in which hectic fever is present.

[1] Birmingham Med. Rev., Jan., 1905. [2] Brit. Med. Jour., Jan. 14, 1905. [3] Jour. Amer. Assoc., 21, 1903. [4] Brit. Med. Jour., Jan. 14, 1905. [5] Progressive Med., Sept. 1904, Vol. III., p. 23. [6] Lancet, Dec. 31, 1904. [7] Birmingham Med. Rev., Feb. 1905. [8] Scottish Med. and Surg. Jour., Jan. [9] Brit. Med. Jour., Jan. 14. [10] Lancet, Jan. 14. [11] Med. Rec., Dec. 3. [12] Brit. Med. Jour., Jan. 14. [13] Lancet, Dec. 31, p. 1827. [14] Edin. Med. Jour., Feb.

(*To be continued.*)

